# A fluorescence approach for an online measurement technique of atmospheric microplastics†

**DOI:** 10.1039/d4ea00010b

**Published:** 2024-03-14

**Authors:** Jürgen Gratzl, Teresa M. Seifried, Dominik Stolzenburg, Hinrich Grothe

**Affiliations:** a Institute of Materials Chemistry TU Wien Vienna Austria hinrich.grothe@tuwien.ac.at; b Department of Chemistry, University of British Columbia Vancouver British Columbia Canada

## Abstract

Microplastic particles in the atmosphere are regularly detected in urban areas as well as in very remote locations. Yet the sources, chemical transformation, transport, and abundance of airborne microplastics still remain largely unexplained. Therefore, their impact on health, weather and climate related processes lacks comprehensive understanding. Single particle detection presents a substantial challenge due to its time-consuming process and is conducted solely offline. To get more information about the distribution, fluxes and sources of microplastics in the atmosphere, a reliable and fast online measurement technique is of utmost importance. Here we demonstrate the use of the autofluorescence of microplastic particles for their online detection with a high sensitivity towards different widely used polymers. We deploy online, single particle fluorescence spectroscopy with a Wideband Integrated Bioaerosol Sensor WIBS 5/NEO (Droplet Measurement Technologies, USA), which enables single particle fluorescence measurements at two excitation wavelengths (280 nm and 370 nm) and in two emission windows (310–400 nm and 420–650 nm). We investigated shredded (<100 μm) everyday plastic products (drinking bottles and yogurt cups) and pure powders of polyethylene terephthalate (PET), polyethylene and polypropylene. For the broad range of typical plastic products analyzed, we detected fluorescence on a single particle level using the WIBS. The online detection can identify particles smaller than 2 μm. In the case of microplastic particles from a PET bottle, 1.2 μm sized particles can be detected with 95% efficiency. Comparison with biological aerosols reveals that microplastics can be distinguished from two abundant pollen species and investigation of the complete fluorescence excitation emission maps of all samples shows that online identification of microplastics might be possible with fluorescence techniques if multiple channels are available.

## Abstract 2

The autofluorescence of airborne microplastics is detected online on a single particle level down to 500 nm particle size.

## Graphical abstract

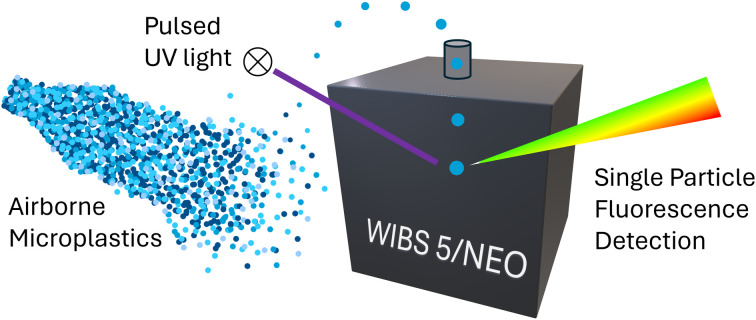

**Environmental significance**

Plastic pollution is a major environmental problem. Especially airborne microplastics and nanoplastics are of concern due to the possibility of inhalation and resulting health risks. So far, little information is available on the concentration, fluxes, and sources of atmospheric microplastics. This is partly because the detection is time consuming and is done solely offline. Here, we demonstrate that microplastics exhibit autofluorescence, detectable on a single particle level with a bioaerosol sensor that gives information about the size and fluorescence of airborne particles in real time. We can discriminate microplastics from pollen grains due to different excitation–emission behavior. With 3D-fluorescence maps, we show that more channels in single particle fluorescence spectroscopy could lead to a reliable and fast online detection method of microplastics.

## 1 Introduction

Synthetic materials have become an indispensable part of our everyday lives. Since the early 20th century, a time considered the beginning of the plastics industry,^1^ the use of polymer products improved our standard of living greatly. In the second half of the 20th century, global plastics production experienced an unprecedented growth and has reached 359 million tons in 2018.^2^ The ever-increasing demand for these products has potential detrimental environmental implications, as a fraction of consumed plastic waste (estimated 22 million tons in the year 2019)^3^ ends up in the environment. Plastic pollution classifies plastics by size, where macroplastics consist of plastic pieces larger than 1 cm, such as polyethylene terephthalate (PET) bottles or high-density polyethylene (HDPE) single-use shopping bags. These can undergo fragmentation due to weathering processes, such as photo-oxidation or mechanical abrasion,^4^ where these macroplastics will decrease in size to form smaller pieces known as secondary microplastics (MPs; <5 mm in size).^5^ Additionally, primary MPs are engineered small plastic particles that are commercially used in products such as cosmetics that will also exist in the environment.^6^

MP particles in soil and aquatic systems impact the environment considerably, for example by changing the behavior and growth of fish,^7^ affecting the structure and function of microbial communities^8^ and may even affect biodiversity in general.^9,10^ Only since the study of Dris *et al.*^11^ in 2015 we know that MPs also occur in the atmosphere. As MPs have been detected not only in urban areas^12–14^ but all around the globe including very remote regions like Mt. Everest,^15^ the high Austrian alps^16^ and Antarctica,^17^ it suggests that these particles reach these remote areas *via* long-range atmospheric transport.

Aerosol particles in the accumulation mode (0.1–1 μm) have the highest residence time in the atmosphere,^18^ and it is suggested that the concentrations of nanoplastics (<1 μm) are orders of magnitudes higher than the concentrations of MPs >10 μm.^19^ Since studies on atmospheric MPs often focus on particles larger than 10 μm,^20,21^ little is known about the actual size distribution and concentration of atmospheric MPs in different environments. Sources of atmospheric MPs include but are not limited to road traffic emissions (tire road wear and tire break wear), sites of plastic waste management and synthetic fibers from clothes.^21–23^ Particles can be (re)suspended from soil and city dust, for example through agricultural activities or traffic.^24^ In the marine environment MPs become airborne through sea spray and bubble burst.^21^ However, due to the limited number of studies on atmospheric MPs, the contribution of these sources as well as the concentration and fluxes into the atmosphere remain highly uncertain.^25,26^ Nevertheless, humans are exposed to airborne MPs and can therefore inhale them, posing potential health risks.^27–29^

Even less is known about the possible implications of these particles on the microphysics of clouds as well as on precipitation and climate related processes,^30^*e.g.*, nanoplastics can potentially act as cloud condensation nuclei^31^ or nucleate ice heterogeneously.^32,33^

The detection of atmospheric MPs is often associated with labor-intensive and time-consuming procedures. First, atmospheric MPs are collected from atmospheric fallout, rain or snow, or *via* active sampling on a filter. Usually, certain steps of sample pre-treatment, *e.g.* filtration and density separation, are necessary to then identify MPs *via* different offline optical or analytical techniques. According to Luo *et al.* (2022),^20^ 42.11% of atmospheric MP studies used optical microscopes for the identification, followed by FTIR (Fourier-transform infrared spectroscopy), SEM (scanning electron microscopy) and Raman spectroscopy. All these methods have their own advantages and disadvantages. The identification of MPs with an optical microscope, for example, is a low-cost method, but highly depends on the skill of the operator identifying them. To avoid a large number of false positive and false negative results, this technique should not be used for particles smaller than 500 μm.^20^ Unless coupled with vibrational spectroscopy, no compositional information about the MP particles can be obtained by optical microscopy. For easier differentiation of MPs and non-polymer particles, some authors used a fluorescence microscope after staining the sample with Nile Red to detect any polymers in the air samples, often before using FTIR.^34^ FTIR and Raman microscopy stand out with the possibility to chemically differentiate between various polymer types.^35,36^ However, FTIR/Raman microscopy is limited by the Abbe diffraction limit, which gives the lower size limit of detectable particles. The Abbe-limit is approximately 5–10 μm for FTIR and 300–500 nm (more realistic is 1 μm if contrast related uncertainties are considered)^37^ for standard Raman microscopy. While the lower limit for particle size is less problematic with SEM, molecular spectroscopic information is not available with electron microscopy. However, coupled with Energy Dispersive X-ray Spectroscopy (EDX), elemental information can be obtained. Besides the number concentrations of atmospheric MPs, some studies use mass spectrometric techniques to evaluate the mass concentration of polymers in the atmosphere.^14,38–40^ Most current detection methods are very time-consuming, as they run offline, and many single particles must be investigated individually. Therefore, only a small number of particles can be examined in one single study. It is very challenging to get real time information about the concentration of MPs in the atmosphere, which currently hinders the estimates of their sources and fluxes, although recent progress has been made using single particle mass spectrometry.^41,42^ Hence, to investigate the distribution and fate of MPs in the air and consequently evaluate health and climate relevant pollution problems, a fast and reliable online measurement technique is required to collect data in real time with high temporal resolution.

In this study, we investigate the possibility of single particle fluorescence measurements as a tool for online detection of airborne MPs. In the last few decades, various studies have reported fluorescence data of polymers. Polymers can be broadly separated into two types by the origin of the photoluminescence emission: Type A and Type B.^43^ The emission of Type B polymers, including PET and polystyrene (PS), arises from the size of the repeat polyaromatic structures (π → π* and n → π* transition).^43^ Solely based on the molecular structure, Type A polymers like polyethylene (PE) and polypropylene (PP) should not emit fluorescence after excitation in the UV-range. However, the fluorescence of PE and PP, even in a very pure form, has been reported already back in the 1960s.^44^ Since then, the nature of the emission was a subject of discussion.^45–48^ Currently the common hypothesis is that the emission is caused by unsaturated carbonyls of the enone and dienone types that are present in the polymers, originating from oxidation processes during synthesis, processing and storage.^49,50^ Recently, a few studies were published in which the autofluorescence of polymers was discussed in the context of MPs. Ornik *et al.* (2020)^51^ showed that different polymers, including PP, PE, PET and PS, can be distinguished from biological materials using fluorescence and Qiu *et al.* (2015)^52^ used the autofluorescence properties of MPs to detect them with a fluorescence microscope prior to FTIR measurements. Monteleone *et al.* (2020)^53^ showed that heat treatment of MPs increased the fluorescence emission resulting in better visibility under a fluorescence microscope. This recent development motivated us to investigate whether autofluorescence as an intrinsic property of MP particles is a promising way to detect them online in the atmosphere. To do so, we use the Wideband Integrated Bioaerosol sensor (WIBS 5/NEO) to characterize single MP particle fluorescence of four different polymers, which we characterized with UV-VIS and FTIR spectroscopy. We further investigate the performance of the WIBS in distinguishing MPs from fluorescent bioaerosols, which are abundant atmospheric fluorescent particles in the coarse size fraction.^54–56^ Further characterization of the MP particles is done with steady-state fluorescence spectroscopy to explore possible improvements towards a reliable online identification method of atmospheric microplastics.

## 2 Materials and methods

### 2.1 Samples and sample preparation

This study investigates the autofluorescence properties of four commonly used types of polymers that are often found in the atmosphere as MPs:^36,57–62^ PET, PP, PE and PS. We used pure samples (>99.9%) of PET, PP and PE as fine powders purchased from Nanochemazone (Canada), denoted by the superscript “a” and more realistic self-fabricated MP samples from everyday packaging products *via* a cryo-milling procedure, denoted by the superscript “b”. The samples were produced from a transparent, light blue PET bottle and a white PP and PS yogurt cup. To fabricate MPs from bulk packaging material, we cleaned the samples of their content in a first step: we used soap and ultra-pure water in the case of the yogurt cups and just water for the PET bottles. Smaller pieces (approximately 5 × 10 cm) were then suspended in acetone for 1 min before rinsing with ultra-pure water (Milli-Q 18.2 MΩ cm at 25 °C). About 500 mg were cut into small flakes (1 to 3 mm^2^) and were put in a grinding jar (volume = 25 ml, stainless steel) with a single grinding ball (diameter = 12 mm, stainless steel). The grinding jar with its content was put into a bath of liquid nitrogen for 5 min before milling with a Retsch MM 400 swing mill for 10 min with a frequency of 30 s^−1^. We conducted this procedure with a nitrogen bath and milling for a total of 10 times. The resulting MP powder was transferred into dark brown glass vials and stored in opaque containers under room temperature. Fig. S1† shows microscopic images of the samples, recorded with a Nikon Eclipse Ci-L microscope (Nikon, Japan). For comparison with biological aerosols, we measured the pollen of the species *Betula pendula* (silver birch) and *Agrostis gigantea* (black bent grass). Table 1 depicts a detailed description of the samples.

**Table 1:** Description of all measured MPs and biological samples. The superscript “a” refers to purchased MP powders and the superscript “b” to powders milled from packaging products. Microscopic images of the MP samples are shown in Fig. S1. The size of the fibres is described by the fibre length

Sample name	Material	Product	Provider	Color	Particle size
PET^a^	PET, (CAS Nr.: 25038-59-9)	“Ultrafine Polyethylene Terephthalate Powder”	Nanochemazone	White	<100 μm
PP^a^	PP, (CAS Nr.: 9003-07-0)	“Fine Polypropylene Powder”	Nanochemazone	White	<30 μm, fibres <150 μm
PE^a^	PE, (CAS Nr.: 9002-88-4)	“Low-Density, Polyethylene Powder”	Nanochemazone	White	<100 μm
PET^b^	Recycled PET	Drinking bottle	Vöslauer	Transparent, light blue	<50 μm
PP^b^	Recycled PP	Yogurt cup	Alpro	White	<100 μm
PS^b^	Recycled PS	Yogurt cup	Vega Vita	White	<50 μm
Pollen A	*Betula pendula*, (Silver birch)	Pollen	Pharmallerga	Yellowish	10–25 μm
Pollen B	*Agrostis gigantea*, (Black bent grass)	Pollen	Allergon	Yellowish	30–50 μm

Chemical characterization with UV-VIS and FTIR-spectroscopy is shown in the ESI (Fig. S2–S4, Table S1†). In short, the FTIR spectra of the self-fabricated powders agree with reference spectra from the literature of the pure substance (Fig. S3†). PP^a^ shows signs of significant aging and oxidation in the FTIR spectrum (Fig. S4†). The UV-VIS spectra of PP^a^ and PP^b^ differ (Fig. S2†), suggesting that UV-VIS absorbing additives are present in PP^b^, which do not absorb in the infrared region.

### 2.2 Single particle fluorescence spectroscopy with WIBS

Fluorescence on a single particle level was measured online using the Wideband Integrated Bioaerosol Sensor 5/NEO short WIBS (Droplet Measurement Technologies, USA). A detailed description of the operating principle of the WIBS can be found elsewhere.^63–66^ In short, the WIBS samples ambient air with a sample flow of 0.3 L min^−1^ and measures the size of single aerosol particles *via* forward light scattering of a 635 nm diode laser. According to the manufacturer, it detects particles from 500 nm to 30 μm diameter. Two xenon lamps with wavelengths of 280 nm and 370 nm are used to excite particles. The fluorescence emission intensity is recorded using two wavebands. The first ranging from 310–400 nm and the second from 420–650 nm. This gives three main channels: FL1 (excitation 280 nm and emission 310–400 nm), FL2 (excitation 280 nm and emission 420–650 nm) and FL3 (excitation 370 nm and emission 420–650 nm). If the emitted light of a particle exceeds the fluorescence threshold in any of the three channels, it is considered to be fluorescent. The fluorescence threshold was determined using forced trigger mode on any day of measurement. In forced trigger mode, the xenon lamps are fired without any particles present. The threshold for every channel is calculated using the mean value of the intensity in the corresponding channel plus three standard deviations. The three main channels can further be combined using ABC analysis, according to Perring *et al.* (2015).^64^ An A particle is a particle that exhibits fluorescence in FL1 only, a B particle exhibits fluorescence in FL2 only, a C particle exhibits fluorescence in FL3 only, an AB particle exhibits fluorescence in FL1 and FL2 only, an AC particle exhibits fluorescence in FL1 and FL3 only, a BC particle exhibits fluorescence in FL2 and FL3 only and an ABC particle exhibits fluorescence in FL1, FL2 and FL3. Fig. 4 shows a scheme of this particle classification. Data analysis was conducted in IGOR pro 9.01 using the WIBS-NEO toolkit (Droplet Measurement Technologies, USA). The setup consists of a small glass vial with an inlet connected to a HEPA filter and an outlet connected to the WIBS. Inside the vial, the samples as well as a small magnetic bar are placed. The vial is put on a magnetic stirrer operated with different “rounds per minute” to create a relatively constant concentration of airborne particles, depending on the sample. The particles are carried to the inlet of the WIBS in-line. The inlet is preceded with an oversized particle trap for the impaction of very big particles. A sketch of the setup is shown in Fig. S5.†

### 2.3 Steady-state fluorescence spectroscopy

In order to get an overview of the general photoluminescence behavior over a wider range of excitation wavelengths, 3D excitation-emission maps (EEMs) of the MP powders were obtained using an FSP920 spectrometer (Edinburgh Instruments, UK), equipped with a 450 W Xe900 xenon arc lamp and an S900 single photon photomultiplier. The instrument was operated in front face geometry using a high precision cell made of two high performance quartz glass slides with a sample depth of 0.2 mm and an associated cell holder (Hellma Analytics, Germany). EEMs were recorded with 5 nm resolution and a dwell time of 0.25 s. Although the EEMs recorded here are obtained from bulk (powder) samples, we wanted to get the representative signals for single aerosol particles. To avoid possible distortion of the emission signal caused by inner filter effects or quenching, powder samples were diluted using quartz sand as a non-fluorescent dilution matrix.^67^ The quartz sand (Carl Roth, Germany) has a purity of >99% and grain sizes <125 μm. A dilution series with dilutions between 10 and 0.01% w/w was conducted.

### 2.4 Further MP characterization

The absorbance spectra of the powder samples were recorded with a Lambda 750 UV-Vis-Spectrometer (PerkinElmer, USA), using the same sample cell as described in Section 2.3 with a 60 mm integrating sphere. The spectrometer uses a tungsten–halogen and a deuterium lamp as a light source and a R928 photomultiplier detector. Scans were conducted with 2 nm resolution from 200–700 nm. Barium sulfate powder was used for auto-zero calibration. FTIR spectra were recorded with an ALPHA II ATR-FTIR Spectrometer (Bruker, Germany) using a DTGS (deuterated triglycine sulfate) detector and a diamond crystal attenuated total refraction unit. For each MP type, four scans were performed for three individual samples. Scans were conducted from 4000 to 400 cm^−1^ with a resolution of 2 cm^−1^.

## 3 Results and discussion

### 3.1 Single particle fluorescence

#### 3.1.1 Fluorescence fractions

**Fig. 1:**
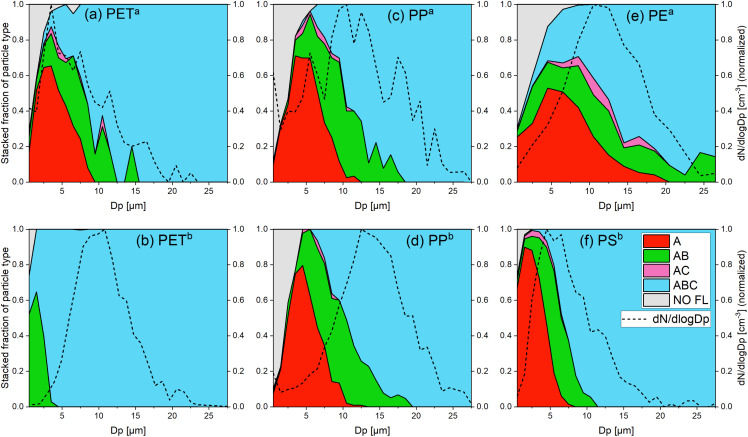
Stacked fraction of the fluorescent particle types and size distribution of all detected particles for (a) PET^a^, (b) PET^b^, (c) PP^a^, (d) PP^b^, (e) PE^a^ and (f) PS^b^. These graphs show the proportion of each particle type (A, AB, AC, ABC and NO FL) in all measured particles of a certain size *D*_p_. The size distributions (d*N*/dlog *D*_p_) were normalized (the maximum was set to 1). The colors refer to the particle types according to the classification in Fig. 4. The gray area shows the fraction of non-fluorescent particles. The line under the gray area is the detection effectivity. For all samples, fluorescence emission on a single particle level could be detected for most of the particles. There is a general trend of channel evolution with the particle size of A → AB → ABC and a few AC particles. Other particle types (*e.g.* B, C and BC) were not recorded.

All studied MPs depicted a characteristic fluorescence signal on a single particle level. Fig. 1 summarizes the stacked fluorescence fractions and size distributions of the plastic samples. The fluorescence fractions of the particle types (A in red, AB in green, AC in pink and ABC in blue) are stacked on top of each other so that the curve under the gray area, which refers to non-fluorescent particles (NO FL), represents the fluorescence detection effectivity. Additionally measured size distributions are added to the fraction plots as dashed lines. In Fig. S6,† the size distributions of all particle types are stacked on top of each other, in order to better see the absolute proportion of particle types per particle size. Fig. 1 depicts a trend that larger particles exhibit fluorescence in more channels. We find a general trend of channel evolution with increasing particle size of A → AB → ABC with a few AC particles for all samples (Fig. 1a and c–f) but PET^b^ (Fig. 1b). PET^b^ particles are mostly of the ABC type and only a fraction of particles <5 μm belong to the AB type, which indicates that PET^b^ particles fluoresce stronger than PET^a^ particles (Fig. 1b). PP^a^ and PP^b^ (Fig. 1c and d, respectively) show a very similar behavior in channel evolution: all particles >20 μm are ABC particles. The smaller particles are allocated to the other channels (A, AB and AC) in a similar way in both cases. PE^a^ (Fig. 1e) shows the least size dependency, as particles >15 μm can still belong to the A and AB types. All PS^b^ particles >12 μm are of the ABC type (Fig. 1f). In general, only a minor fraction of the particles showed no fluorescence above the WIBS size threshold (Table 2). In the case of PET^b^ and PS^b^ almost 100% of the particles emit measurable fluorescence. For the other samples, the fluorescence fraction ranges from 87.5% for PE^a^ to the lowest value of 62.6% for PP^a^. The total fluorescence fraction depends on the form of the size distribution, since smaller particles exceed the fluorescence threshold less often. Therefore, the fluorescence cut-off diameters *D*_50_ and *D*_95_ were calculated (defined as the particle diameter at which 50% and 95% of the particles show a fluorescence signal in at least one channel, respectively). The values for *D*_50_ range from below the detection limit for PET^b^ and PS^b^ to the highest value of 2.4 μm for PP^a^ (Table 1). The lowest value of *D*_95_ is 1.2 μm for PET^b^ and the highest is 5.7 μm for PE^a^. Therefore, almost all MP particles emit measurable fluorescence above 5 μm, demonstrating an excellent sensitivity of the WIBS towards atmospheric MP detection above that size.

**Table 2:** Values of the fraction of fluorescent particles, *D*_50_ and *D*_95_ (particle diameter at which 50% and 95% of particles are detected as fluorescent) ordered in increasing value of *D*_95_^a^

Sample name	FL fraction [%]	*D*_50_ [μm]	*D*_95_ [μm]
PET^b^	99.9	bld	1.2
PS^b^	98.6	bld	1.4
PET^a^	75.4	1.3	3.4
PP^b^	79.1	2.4	4.8
PP^a^	62.6	2.4	4.9
PE^a^	87.5	2.0	5.7

a bld = below limit of detection.

#### 3.1.2 Relative fluorescence intensities

**Fig. 2:**
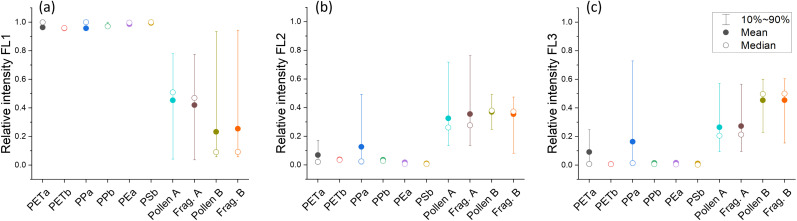
Relative fluorescence intensities (value of each channel divided by the sum of all channels) for (a) FL1, (b) FL2 and (c) FL3 for all MP samples and fragments and whole pollen grains of two different pollen species, Pollen A (silver birch) and Pollen B (black bent grass). MP samples dominate in the FL1 channel, whereas pollen samples have a broader distribution. Only in the channels FL2 and FL3, PET^a^ and PP^a^ show an overlap of 10–90 percentiles (represented by whiskers) with the pollen samples. Note that only particles that show fluorescence in a certain channel are considered. Therefore, whiskers in FL2 and FL3 can be wider than in FL1 because fewer particles show fluorescence in those channels.

The absolute fluorescence intensity depends strongly on the particle size. We find an increase of fluorescence intensity with increasing particle size for all samples except of PE^a^. The intensity–size relationship is shown in the ESI in Fig. S7.† In Fig. S8,† the absolute fluorescence intensities of the three channels FL1, FL2 and FL3 of all samples are plotted against each other for certain size ranges, clearly showing the differences between Pollen A, Pollen B, PET^b^ and the rest of the MP samples. In this manner, it is possible to differentiate the pollen samples and the MP samples, as well as PET^b^ and all other MP samples in certain size ranges. However, to avoid a bias due to this strong size-dependency of the absolute fluorescence signal, especially as the atmospheric size-distribution of MPs remains largely unknown to date, we calculated the relative fluorescence values for the three fluorescence channels (absolute value for each channel divided by the sum of the three channels). We compared them with the values for pollen samples (Fig. 2). One key element in using online single particle fluorescence techniques to detect MP particles is to discriminate MPs from other fluorescent aerosols. In the scope of this study, we compared MPs with two different kinds of pollen grains, as bioaerosols and especially pollen grains are the dominant fluorescent particles above 5 μm, *i.e.* the range where the WIBS has a very high sensitivity towards MPs. The size distribution of the pollen samples shows two distinct peaks with maxima at 4 and 27 μm for Pollen A and 8 and 27 μm for Pollen B. Therefore, every pollen sample is treated as two different samples: pollen fragments (≤15 μm) and pollen (>15 μm). Stacked fraction plots together with the size distribution of the pollen samples can be seen in Fig. S9.† For all MP samples, the fluorescence in channel FL1 dominates (Fig. 2a). Therefore, the FL1 fraction is close to 1, whereas FL2 and FL3 fractions are very small. An overlap of the percentiles between the group of the MP samples and the pollen samples is measured only for PET^a^ and PP^a^ in the FL2 and FL3 channels. For the other types of MPs, a clear distinction between MPs and pollen can be made in all channels, and, especially when combined, this is a powerful tool to differentiate between MPs and other fluorescent atmospheric particles above 5 μm such as pollen. In general, fluorescent interfering particles in the atmosphere complicate the interpretation of data obtained by any online biological particle sampler. Several aerosol particles have been identified as interfering particles, such as diesel soot, ash, cotton fibers and others.^66^ However, most interfering particles fluoresce with a lower intensity than biological particles. Therefore, when setting the fluorescence threshold to 9 standard deviations over the forced trigger mean value compared to 3 standard deviations, most biological particles are still classified as fluorescent, while interfering particles are mostly classified as non-fluorescent.^66^ In Fig. S10 in the ESI,† we show that increasing the threshold to 9 sigma has only a minor effect on the detection of MP samples: The cutoff diameter *D*_50_ is shifted to higher diameters (still below the limit of detection for PET^b^ and between 1.3 and 5.2 μm for the other samples).

Certainly, measurements under real life conditions would generate much more complex data sets. Not only more types of interfering particles are present in the atmosphere, but of course, the diversity of biological particles in the atmosphere is more complex than can be described by two pollen species. However, our results demonstrate that single particle fluorescence is intense enough to be measured by commercially available bioaerosol sensors like the WIBS 5/NEO, even for polymers without any aromatic structures.

Fluorescence was detected on a single particle level down to 500 nm particle size (lower size limit of the WIBS) with an effectivity of 50% for two of the MP types, whereas 2 μm is the highest value of *D*_50_. These values are in the range of the size limitations of the offline techniques (approximately 10 μm for FTIR and 1 μm for Raman) and therefore, the significant advantages of an online method using fluorescence like high time resolution and availability of real time data are not accompanied by restrictions of a larger particle size detection limit compared to existing methods. While we showed that using the WIBS 5/NEO we can distinguish between MPs and two types of pollen grains using relative fluorescence values, it was not possible to differentiate between different types of polymers. This is a clear disadvantage compared to FTIR and Raman, where the chemical information is used to assign a polymer type to MP particles. The reason for the low selectivity of the WIBS is that only using three fluorescence channels gives little information on the complex fluorescence behavior of the polymers. Therefore, we conducted 3D fluorescence EEMs to get an overview of the general emission behavior and to explore future improvements on this online detection technique.

### 3.2 Excitation–emission maps

**Fig. 3:**
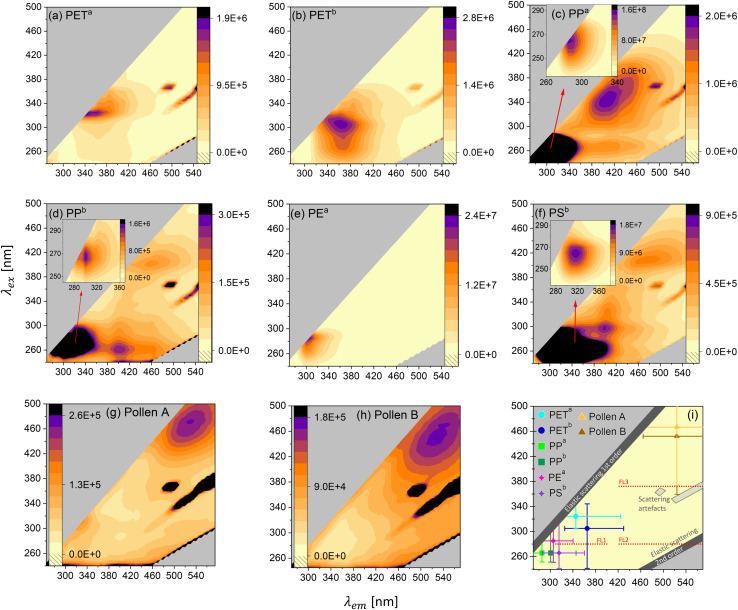
Excitation–Emission Maps (EEMs) of all samples investigated in this study. To avoid distortion of the emission signal due to the inner filter effect for emission and quenching, we diluted the samples with non-fluorescent quartz sand. (a) PET^a^ (dilution: 0.90% w/w), (b) PET^b^ (dilution: 0.90% w/w), (c) PP^a^ (undiluted), (d) PP^b^ (undiluted), (e) PE^a^ (undiluted), (f) PS^b^ (undiluted), (g) Pollen A (dilution: 0.70% w/w) and (h) Pollen B (dilution: 0.08% w/w). The color code represents the intensity of the emitted light in arbitrary units. (i) The most intense excitation–emission maximum of all MP types and pollen grains. The bars represent the regions, where at least 25% of the intensity of the maximum is detected. FL1 and partly FL2 and FL3 of the WIBS are depicted.

Getting a better understanding of the general photoluminescence behavior of the samples helps to differentiate MPs from biological materials and other carbonaceous particles and potentially also enables MP differentiation. Fig. 3a–h depict the EEMs of PET^a^, PET^b^, PP^a^, PP^b^, PE^a^, PS^b^, Pollen A and Pollen B. Fig. 3i summarizes the (most intense) excitation–emission maximum of the MP and pollen samples. All samples show autofluorescence with different maxima. In addition to the fluorescence emission, all maps but (e) show instrument related scattering artifacts. These signals occur in all solid samples with the used instrument. Further, we find that the EEMs change with the rate of dilution for PET^a^, PET^b^ and pollen samples. Fig. S11† shows the maps of PET^a^, PET^b^, Pollen A and Pollen B in an undiluted form and in various dilutions. PET^a^ (Fig. 3a) shows a maximum at an excitation wavelength of *λ*_ex_ = 325 nm and an emission wavelength of *λ*_em_ = 345 nm, whereas PET^b^ (Fig. 3b) has the strongest signal at *λ*_ex_ = 305 nm and *λ*_em_ = 365 nm. The intensity of PET^a^ and PET^b^ at their individual excitation–emission maxima is very similar. However, PET^b^ has stronger emission towards smaller excitation wavelengths compared to PET^a^. This leads to a higher fluorescence intensity of PET^b^ in the WIBS channel FL1 (see Fig. S7a†). Even though the absorption spectra of PET^a^ and PET^b^ only differ in the visible range (due to the blue color of PET^b^, see Fig. S2†), the excitation spectrum (excitation at a certain emission wavelength) of PET^b^ is higher at lower wavelengths, suggesting that additives to the PET bottle in combination with the existing absorbers cause higher fluorescence at lower wavelengths.

The samples PP^a^ (Fig. 3c) and PP^b^ (Fig. 3d) both show three distinct excitation-emission maxima. For PP^a^ the main maximum (*λ*_ex_ = 265 nm and *λ*_em_ = 285 nm) is about two orders of magnitudes more intense than the other two maxima at around 400 nm emission. The main maximum for PP^b^ (*λ*_ex_ = 265 nm and *λ*_em_ = 300 nm) is more pronounced by a factor of about 10 compared to the other maxima at higher emission wavelengths. PP^a^, although less absorbent in the UV-VIS range compared to PP^b^ (Fig. S2†), shows 100-times higher fluorescence at the main maximum, probably an effect caused by oxidation/aging, visible in the FTIR spectrum (Fig. S4†). PS^b^ (Fig. 3f) also shows three distinct maxima. The most intense signal is at *λ*_ex_ = 265 nm and *λ*_em_ = 315 nm and is more intense by a factor of 20 and 30 compared to the other two maxima. PE^a^ (Fig. 3e) shows one distinct maximum at *λ*_ex_ = 285 nm and *λ*_em_ = 305 nm. The two pollen samples exhibit the strongest emission at much higher wavelengths than the MPs. This supports the difference between pollen and MPs measured with the WIBS. In Fig. 3i, the most intense maximum of the MPs and the pollen samples is plotted together with the WIBS channel FL1 (and parts of FL2 and FL3). For all MP samples, the emission intensity peaks between 285 nm and 365 nm and therefore is always in the UV-A and B range.

An online fluorescent particle detector, where the UV-A and B range is covered with higher resolution (more channels), could thus lead to better discrimination between different MP types. For example, in addition to the existing excitation wavelength of 280 nm of the WIBS, an excitation at 260 nm would cover the maxima of PP and PS and an excitation of about 330 nm would be in the region of the PET maxima. Combining with adding more emission channels, starting at small Stokes shifts (shift from the excitation to the emission wavelength) of 20 nm, could lead to improvements in the discrimination of various MPs. A smaller Stokes shift is a common property among the polymeric materials considered here. Fluorescence in the UV-A and UV-B range in combination with a small Stokes shift seems to be rare in biological fluorophores, pollen and other interfering particles.^68,69^ Hence, small Stokes shift emission channels would substantially contribute to the discrimination of polymers, biological and other interfering particles. Last, fluorescence lifetime (the time it takes for the excited electron to reemit the photon) measurements should be explored since fluorophores emitting in similar regions might have different lifetimes in the excited state, potentially being the key to differentiate between different types of polymers. Altogether, our results show that with some improvements on fluorescence information, online MP detection and identification is possible and should be further investigated in the future, while complementary techniques such as holographic images might further help to differentiate MPs from biological particles, as the latter often have distinct morphologies.

## 4 Conclusions

In this paper, we show that MP particles belong to the subset of fluorescent aerosols and that we can measure fluorescence emission at the single particle level. This property opens the possibility to detect airborne MPs online and consequently analyze their distribution and fate in the atmospheric environment, yet, with the challenge to efficiently discriminate between polymer types. This study was designed as a first proof of concept that, in general, bioaerosol sensors like the WIBS 5/NEO can detect the fluorescence signal of single MP particles of a variety of common polymers. We were able to differentiate between MPs and pollen grains (*Betula pendula* and *Agrostis gigantea*, which serve here as representatives for tree and grass pollen) using the relative fluorescence values of the WIBS 5/NEO. However, we also showed that the low resolution of three fluorescence channels of the WIBS 5/NEO is not sufficient to distinguish different types of MPs as they produce very similar signals integrated over the wide emission bands of the WIBS. Through the EEMs we show the differences in the emission behavior when looking at higher emission wavelength resolution. Designing an instrument with specific excitation and emission channels targeting the areas where polymers show high fluorescence could lead to a satisfactory discrimination between different polymer types. Polymers are used in so many areas of our everyday life that it is expected that one type of polymer will be mixed with a variety of additives to produce the desired properties. In future studies, it should be explored to which extent additives affect the photoluminescence performance by characterizing MPs of one polymer type from a variety of products. However, the fact that pure polymers without additives show fluorescence is very promising and clearly guides the way towards an online measurement technique for atmospheric MPs.

## Author contributions

JG performed the measurements. JG analyzed the data. JG, TMS, DS and HG contributed to the scientific discussion and interpretation of the results. JG wrote the manuscript draft. TMS, DS and HG reviewed and edited the manuscript. HG purchased the WIBS as part of his appointment.

## Conflicts of interest

There are no conflicts to declare.

## Appendix

**Fig. 4:**
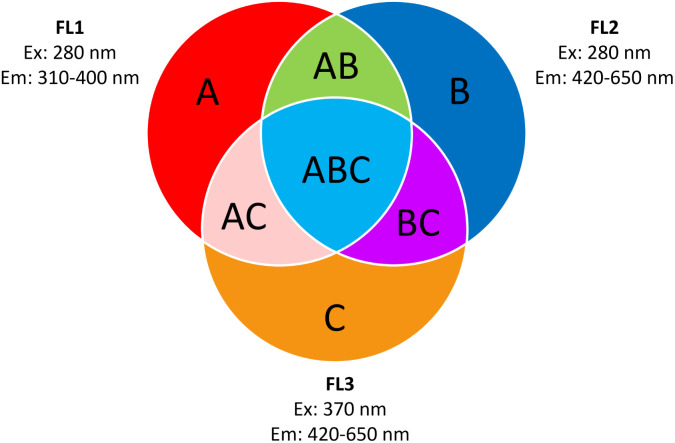
ABC-particle classification according to Perring *et al.* (2015).^64^ (A) Fluorescent particles detected in FL1 only. (B) Fluorescent particles detected in FL2 only. (C) Fluorescent particles detected in FL3 only. (AB) Fluorescent particles detected in FL1 and FL2 only. (AC) Fluorescent particles detected in FL1 and FL3 only. (BC) Fluorescent particles detected in FL2 and FL3 only. (ABC) Fluorescent particles detected in FL1, FL2 and FL3. Graph recreated from Savage *et al.* (2017).^66^

## Supplementary Material

EA-004-D4EA00010B-s001
