# Supplementary material for: A fluorescence approach for an online measurement technique of atmospheric microplastics
Source: Environ Sci Atmos. 2024 Mar 14;4(6):601–10. doi: 10.1039/d4ea00010b (PMC11170559; doi:10.1039/d4ea00010b)
Supplement: EA-004-D4EA00010B-s001 [file EA-004-D4EA00010B-s001.pdf]

Electronic Supplementary Material (ESI) for Environmental Science: Atmospheres.
This journal is © The Royal Society of Chemistry 2024

Supplement to "A fluorescence approach for an online measurement technique of atmospheric microplastics"

*Jürgen Gratzl[1], Teresa M. Seifried[2], Dominik Stolzenburg[1], Hinrich Grothe[1]**

[1]Institute of Materials Chemistry, TU Wien, Vienna, Austria

[2]Department of Chemistry, University of British Columbia, Vancouver, British Columbia, Canada

*Corresponding author. hinrich.grothe@tuwien.ac.at

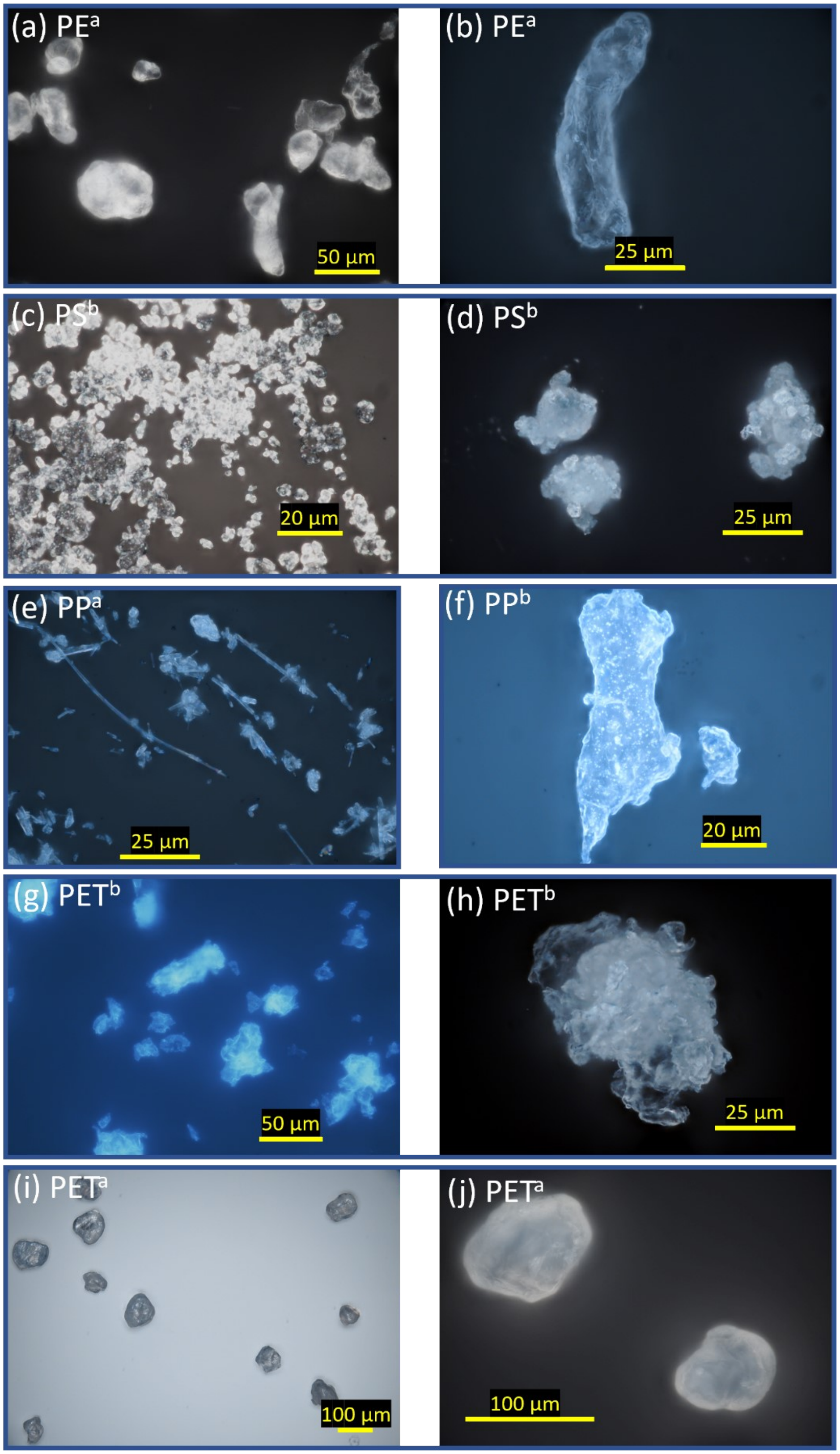

Figure S1. Bright field microscopic pictures of MP samples.

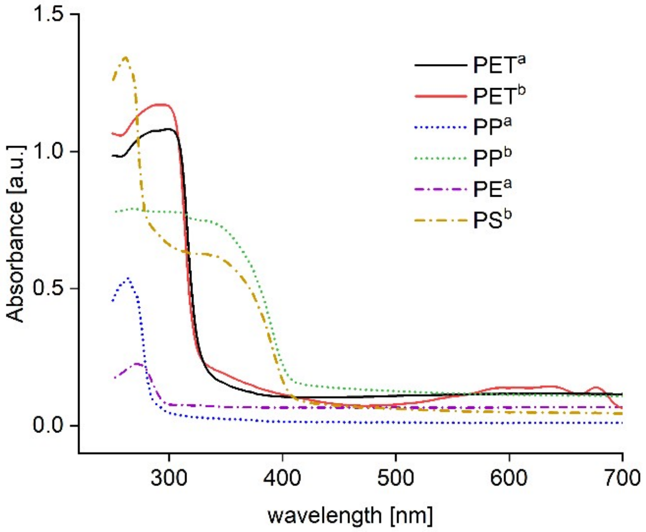

Figure S2. Absorbance spectra of the MP samples. PET^b only differs significantly from PET^a in the visible range, due to the blue color of the PET bottle used to fabricate PET^b. The spectrum of PP^b and PP^a differ as PP^b absorbs up to over 400 nm, whereas the absorption of PP^a ends at 300 nm. UV-absorbent additives added to the PP cup used to fabricate PP^b is probably responsible for this.

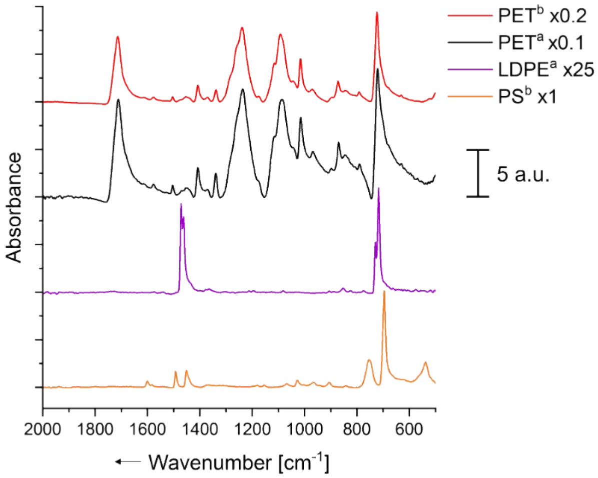

Figure S3. ATR-FTIR spectra of PET^b, PET^a, PE^a and PS^b. Spectra were normalized by setting the CH_2 asymmetric stretch band (~ 2919 cm^{-1} for PE^a and PS^b and ~ 2960 cm^{-1} for both PET samples) to one. Both PET spectra show the typical bands associated with pure PET and no additional bands are found (1). Also PS^b and PE^a show no additional bands to the reference spectra of the pure substances (2,3).

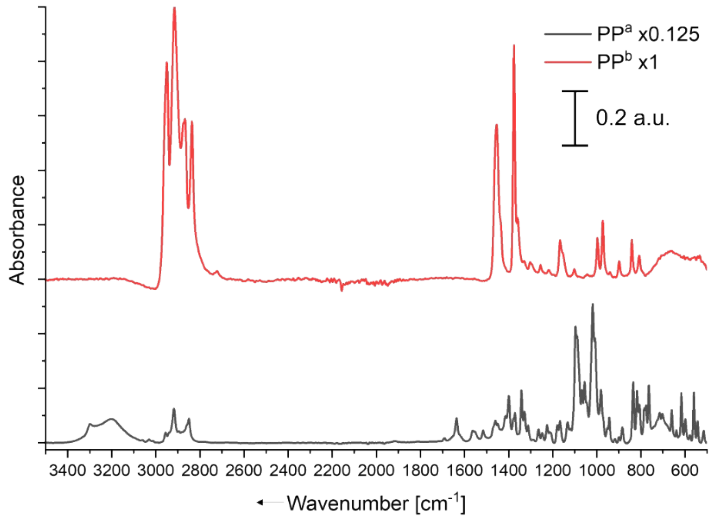

Figure S4. ATR-FTIR spectra of PP[a] and PP[b]. Spectra were normalized by setting the CH_2 asymmetric stretch band (~ 2919 cm^{-1}) to one. PP[b] shows no significant deviation to reference spectra of pure PP (3), suggesting that UV-absorbing additives are not absorbent in the IR region. PP[a], although > 99.9 % pure (according to manufacturer), shows many additional bands in the spectrum which indicate strong oxidation and aging. For instance, the formation of alcohols, as suggested by the broad O-H band (> 3000 cm^{-1}) and the characteristic C-O stretching bands (~ 1000 - 1100 cm^{-1}), and possibly the formation of carbonyls (~ 1640 cm^{-1}).

Table S1. Assignment of important bands of the FTIR spectra of the MP samples

**Sample**	**Band [cm^{-1}]**	**Assignment**
PET[a], PET[b]	1714	C=0 stretch
	1239	C-C-O stretch (aromatic ester)
	1093	O-C-C stretch (aromatic ester)
PE[a]	1472, 1462	CH_2 bend
	730, 718	CH_2 rock
PS[b]	1600, 1493	C=C stretch
	755, 697	C-H bend (aromatic out-of-plane)
PP[b]	1454	CH_2 bend
	1375	Symmetric CH_3 deformation
	1167, 998, 973, 841	Characteristic vibrations of Isotactic PP

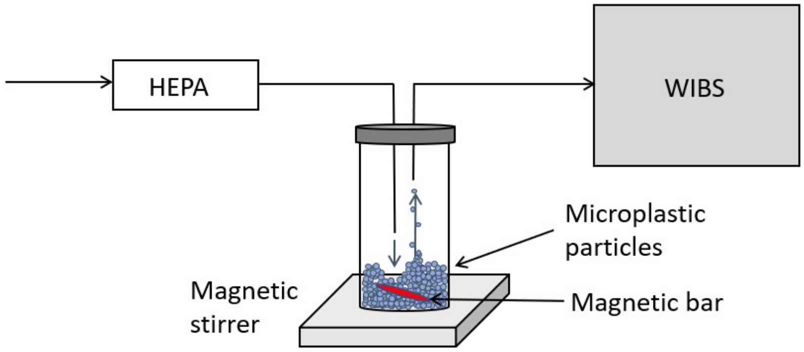

Figure S5. Experimental setup for WIBS measurements.

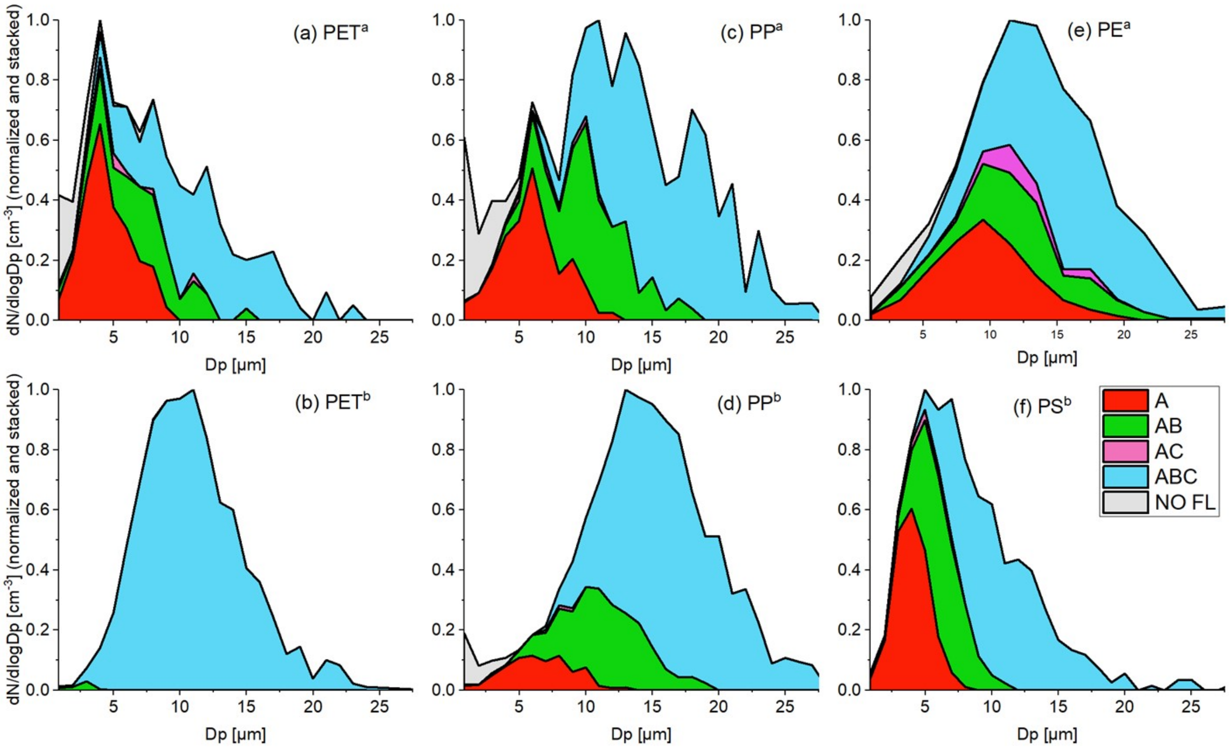

Figure S6. Stacked size distributions of all measured particle types. This figure contains the same information as Figure 1, however, the absolute contribution of the particle type per measured particle size is depicted in a clearer way.

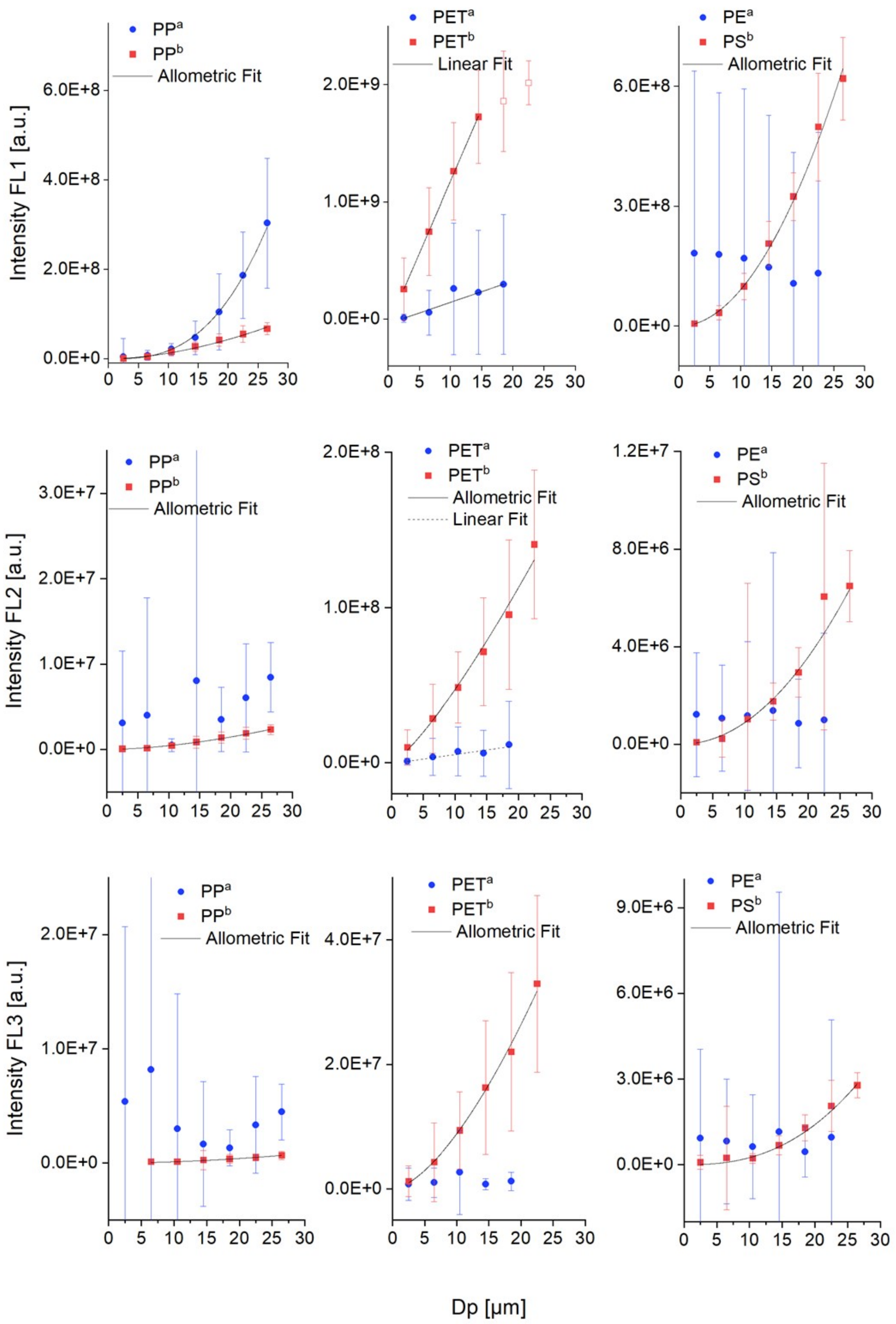

Figure S7. Fluorescence Intensity of channels FL1 (a), FL2 (b) and FL3 (c) depending on particle size. Absolute fluorescence values are calculated for seven different size bins of 4 µm channel width. The values are threshold corrected (i.e. raw intensity value minus threshold value). Values for size bins in which less than 10 particles are recorded are not reported here. Error bars represent the standard deviation. The two datapoints for PET[b] associated with the biggest particle sizes (empty squares in (b)) are excluded from the fit since more than 50 % of the particles exhibit emission in the overflow of the instrument. We find an increase of fluorescence intensity with increasing particle size for all samples except of PE[a].

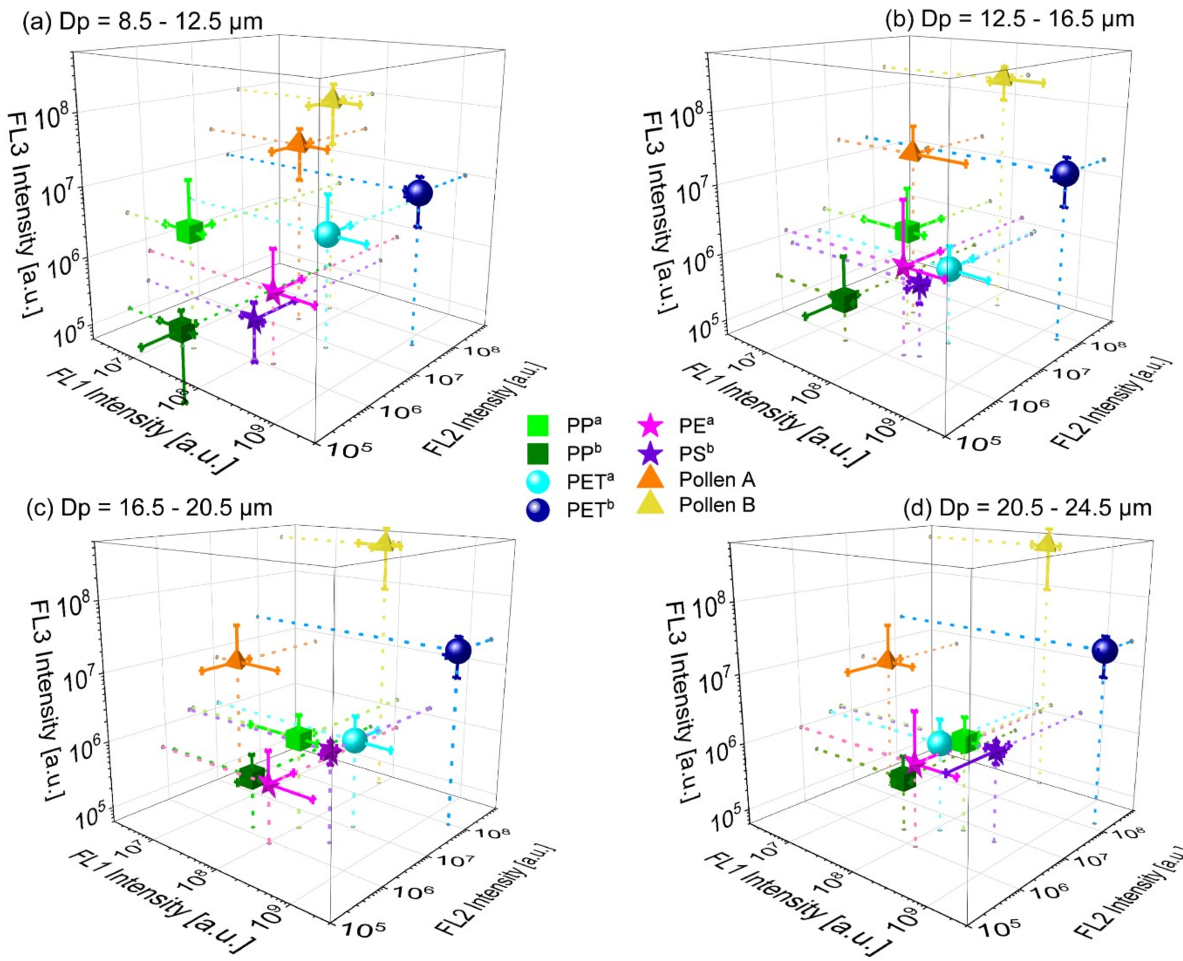

Figure S8. 3D-plots of the absolute fluorescence intensities of the channels FL1, FL2 and FL3 of all samples for the particle size ranges (a) 8.5 – 12.5 µm, (b) 12.5 – 16.5 µm, (c) 16.5 – 20.5 µm and (d) 20.5 – 24.5 µm (d). Error bars represent the standard deviation. The dotted lines are added to guide the eyes. The data plotted here is the same as in Figure S7 for a certain size bin with additional data from the pollen samples. However, plotting all samples in one graph allows a direct comparison with all samples. One can clearly see the difference of the pollen samples compared to the MP samples. Especially in FL2 and FL3, the signal is higher in all size ranges except that PET^b shows a higher FL3 intensity than Pollen A in (c) and (d). The high intensity of PET^b in all three channels, allows it to distinguish PET^b from all other samples in the shown size ranges. The remaining MP samples lie relatively close to one another, resulting in an overlap of the standard deviations in most cases. Exceptions are for example PP^a, PP^b and PS^b, which can all three be distinguished in the size range 20.5 – 24.5 µm by their FL1 intensity (compare with Figure S7a).

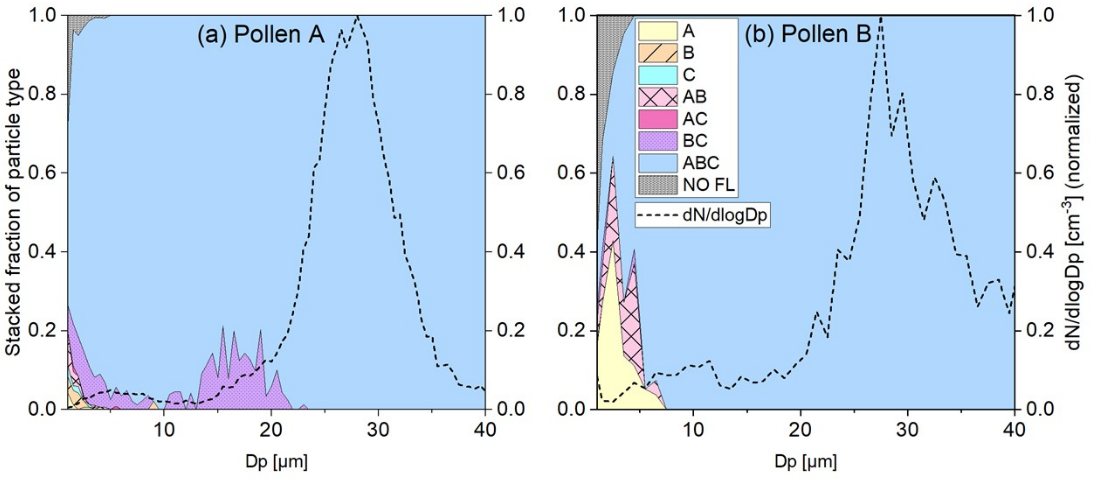

Figure S9. Stacked fraction of particle types and size distribution for (a) Pollen A and (b) Pollen B. For both Samples two peaks can be observed. We name the first peak (≤ 15 µm) pollen fragments and the second (> 15 µm) pollen grains.

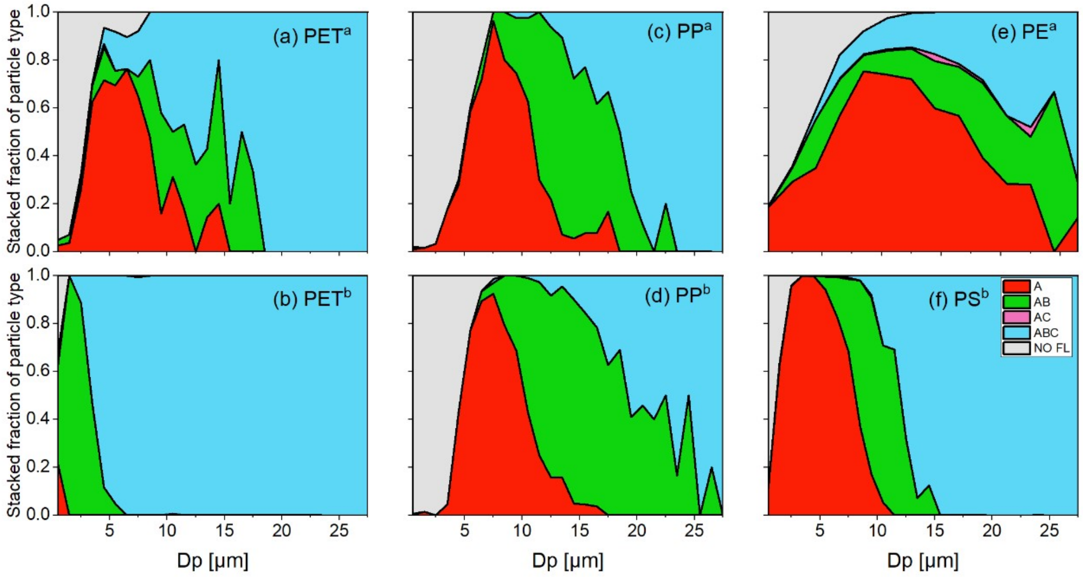

Figure S10. Stacked fractions of particle types. The fluorescence threshold was calculated by adding 9 standard deviations to the mean forced trigger value (compared to 3 standard deviations in the manuscript). The higher threshold leads to a higher D_{50} cutoff diameter (diameter at which 50 % of all

particles fluoresce above the threshold). The value of D_{50} is 3.0 µm for (a) PET[a] , below detection limit for (b) PET[b], 5.2 µm for (c) PP[a], 4.7 µm for (d) PP[b], 3.7 µm for (e) PE[a] and 1.3 µm for (f) PS[b].

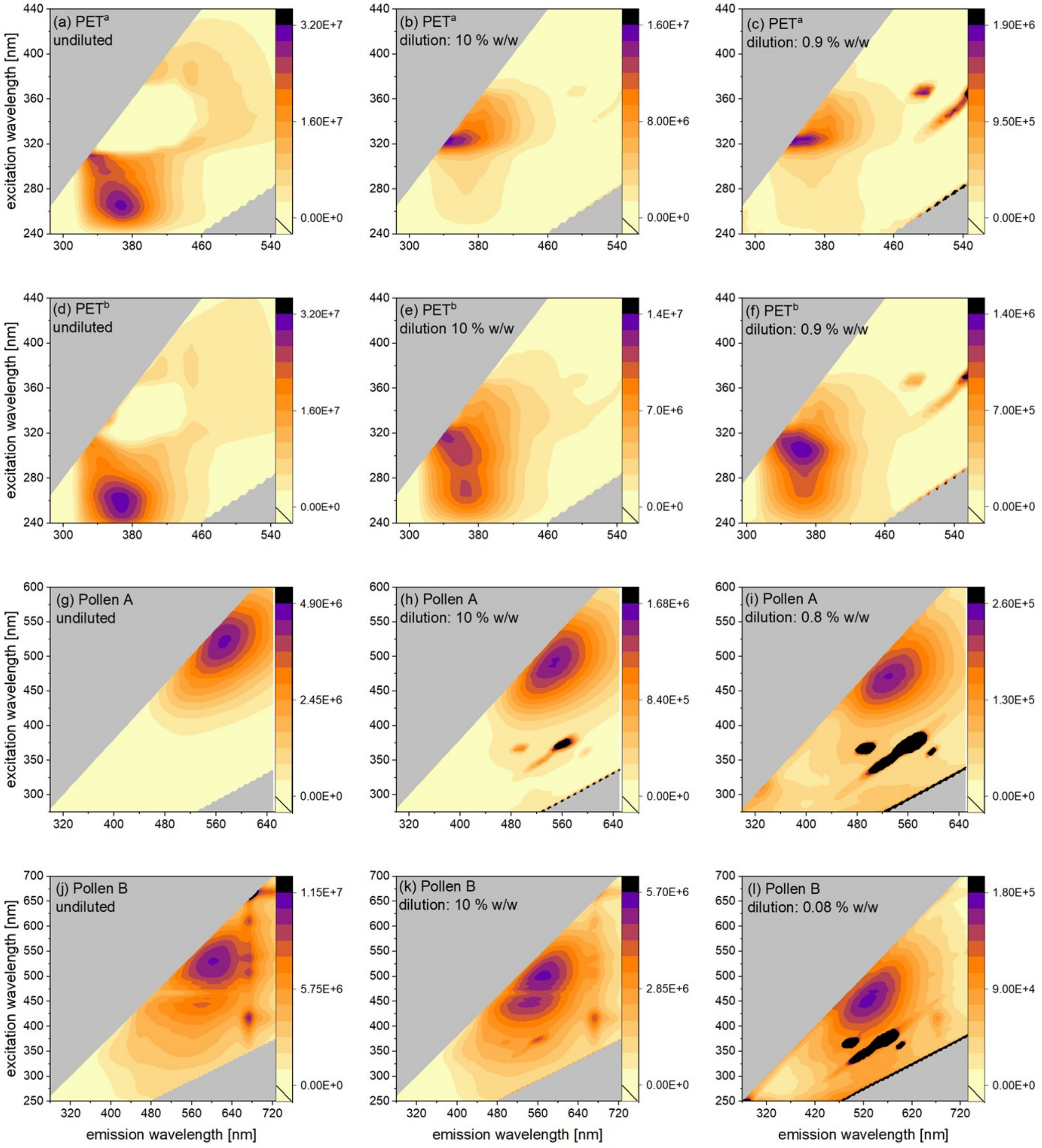

Figure S11. Change of the 3D-Fluorescence spectrum of PET[a], PET[b], Pollen A and Pollen B with rate of dilution. The color code gives fluorescence intensity in arbitrary units. For PET[a], the 10 % dilution (b), for PET[b] and Pollen A the ~0.9 % dilution (f and i) and for Pollen B the ~0.08 % dilution (l) is considered the non-suppressed signal (i.e. no inner filter effect/quenching). Therefore, except for PET[a] these dilutions are shown in Figure 3. For comparison between PET[a] and PET[b] both samples are presented as ~0.9 % dilutions

in Figure 3. For all other MP samples there was no shift in the excitation-emission maximum depending on the dilution observed. Therefore, the signal of the undiluted samples is considered the non-suppressed fluorescence signal.

References

1. V. Borjanović, L. Bistričić, I. Pucić, L. Mikac, R. Slunjski, M. Jakšić, G. McGuire, A. Tomas Stanković and O. Shenderova, *J Mater Sci* 2016, *51*.

2. J. V. Gulmine, P. R. Janissek, H. M. Heise, L. Akcerud, *Polym Test* 2002*, 21*, 557-563.

3. B. Smith, *Spectroscopy* 2021, *36*, 22-25.
